# A flow cytometric assay for the quantification of MSC lysis by peripheral blood mononucleated cells

**DOI:** 10.1016/j.heliyon.2021.e06036

**Published:** 2021-02-01

**Authors:** Katia Chieregato, Martina Bernardi, Alberta Alghisi, Rosaria Giordano, Lorenza Lazzari, Omar Perbellini, Mario Rassu, Marco Ruggeri, Giuseppe Astori

**Affiliations:** a Advanced Cellular Therapy Laboratory, Haematology Unit, Vicenza Hospital, Italy; b Immunohematology and Transfusion Medicine Service, Vicenza Hospital, Italy; c Laboratory of Regenerative Medicine - Cell Factory, Department of Transfusion Medicine and Hematology, Fondazione IRCCS Ca’ Granda Ospedale Maggiore Policlinico, Milano, Italy; d Department of Microbiology, Vicenza Hospital, Italy; e CORIS Veneto - Consorzio per la Ricerca Sanitaria, Padova, Italy

**Keywords:** Mesenchymal stromal cell, Absolute count, Cytotoxicity, Graft versus host disease, Apoptosis

## Abstract

Mesenchymal stromal cells (MSC) are attractive candidates for the treatment of acute graft versus host disease (aGvHD) or autoimmune disorders. However, mechanisms of MSC recognition remain unclear and there are evidences that MSC are not totally immunoprivileged. Data suggest that MSC undergo apoptosis after infusion in presence of cytotoxic cells and their death could drive immunosuppression. In GvHD patients, that activity was associated with clinical response. It is mandatory to develop an *in vitro* potency testing predictor of the "in vivo" response to the therapy.

We describe a flow cytometric assay based on differential immunostaining of target and effector cells where BM MSC are enumerated with fluorospheres to determine the loss of target cells after co-culture with PB MNC.

6/13 (46%) of BM MSC lots were lysed by PB MNC and the lysis was proportional to the E/T cell ratio.

The method overcomes the problems linked to the use of dyes or radioactive, evidencing the limitations linked to the use of a single vital dye and proposing a precise gating strategy based on absolute cell counts where cells are left untouched. The assay is easy and could be used to predict the response of the patients to the therapy.

## 1 Introduction

Mesenchymal stromal cells (MSCs) are non-hematopoietic multipotent stem cells that can be isolated from adult or perinatal tissues [1, 2] and differentiated into mesodermal lineages [3, 4].

Early *in vitro* observations that *ex-vivo* expanded MSCs were able to inhibit T cell proliferation supported their use as immune modulators [5, 6, 7]. For several years, MSCs have been considered to be immunoprivileged due to a low expression of HLA class I, lack of HLA class II or co-stimulatory molecules like B7-1, B7-2 or CD40 [8]. Moreover, MSCs seemed to be unable to activate allogeneic lymphocytes in a mixed culture leading to the hypothesis that MSCs could potentially overcome the HLA barriers in any clinical application using a single potential universal MSCs donor [9, 10, 11]. There is evidence that allogeneic MSCs can be lysed by activated natural killer (NK) cells [12, 13, 14, 15], cytotoxic CD8+ T cells [15] and by cytokine-induced killer cells (CIK) [16]. Additionally, MSCs can induce memory T cells [17, 18] and lead to the formation of alloantibodies [19, 20]. *In vivo*, the vast majority of infused MSCs are entrapped in the lungs [21] and subsequently lysed by host cytotoxic cells. For all these reasons, MSCs were defined “immunoevasive” rather than “immunoprivileged” [22] since they do not completely escape the attack of host lymphocytes [23].

MSC immunomodulation made them attractive candidates as therapeutic agents for diseases where the immune system is involved, such as autoimmune disorders [24] and acute graft versus host disease (aGvHD) after allogeneic hematopoietic stem cell transplantation on patients refractory to conventional therapies [25].

The safety of third-party MSC infusion has been assessed by several groups (reviewed in [26]) but surprisingly, despite strong *in vitro* evidences, infusion of allogeneic MSCs in GvHD patients produced contradictory results. Indeed, we have to emphasize that how a MSC batch is chosen/selected/assigned for a specific recipient is not well defined yet.

It is possible that the causes of these variable responses to MSC therapy could be attributed to the patient's characteristics (for which a reliable biomaker does not yet exist) or to the features of the infused MSC batch, which is in turn related to the tissue of origin and the ex-vivo expansion procedures [27, 28].

Therefore, the selection of the most effective batch of MSCs could help to make this promising therapy more effective.

In this context, Dazzi and colleagues have demonstrated that MSC death could drive immunosuppression, consequently the cytotoxic activity against MSCs by host immune system could become a requirement for their clinical response [29].

If the MSC efficacy *in vivo* is guided by their ability to go into apoptosis once recognized by the host's lymphocytes, their functionality can be estimated in a cytotoxicity assay where MSCs and host lymphocytes are co-cultured.

The use of radioisotopes, as Chromium 51 (^51^Cr), have been considered for a long time the reference method to quantify cytotoxicity despite of its several disadvantages and functional limitations. The Chromium is hazardous to health, has a short half-life time, the assay is not interpretable in the presence of a spontaneous release and it underrates the cytotoxicity at low E:T ratios.

This limitation induced us to test two nonradioactive probes: calcein AM (CAM) and 3,3′-dioctadecyloxacarbocyanine (DIOC18). Calcein AM is a non-radioactive lipid soluble fluorogenic esterase substrate that passively crosses the cell membrane and inside the cell is converted into a green fluorescent product named calcein that is retained in the undamaged cells. DIOC18 is an amphiphilic green fluorescent membrane dye member of the carbocyanine family used for cell tracking.

In our experience, both probes showed an unstable integration in BM MSCs and the leakage of the dyes to neighboring cells was the main problem hindering the discrimination of target and effector cells (data not shown).

The problem of the discrimination between target and effector cells recurred when we quantified the dead MSCs with a viability marker (7AAD). Dead cells of both cell populations gave nonspecific signals, leading to the underestimation of the cytotoxic effect in case of a complete destruction of the target by the effector cells.

To overcome the critical issues described above, we describe a quantitative single platform assay based on differential immunostaining of MSCs with CD105 and of the PB MNCs with CD45 in combination with the viability marker 7AAD and Flow Count fluorospheres.

## 2 Materials and methods

### 2.1 Isolation and culture of BM MSC

Total nucleated cells (n = 13) were isolated from the washout of discarded filters used for bone marrow (BM) collection. We chose this source since BM remains a widely recognized source of MSC for clinical use. The procedure was approved by the Comitato Etico Per le Sperimentazioni Cliniche della Provincia di Vicenza (Act 40/09 of 16.12.2009). Informed consent was obtained from patients involved in the study. After two washing steps with 200 ml saline solution and centrifugation at 2 000 RPM for 10 min, the collected nucleated cells were seeded in toto at the density of 1 × 10^5^ cells/cm^2^ in low-glucose Dulbecco's modified Eagle's medium (DMEM) with GlutaMAX™ and pyruvate (Gibco, Invitrogen, Carlsbad, USA) supplemented with 10% fetal bovine serum (FBS, Qualified Australian, Gibco, Invitrogen) and 1% penicillin/streptomycin (Sigma-Aldrich, St Louis, USA). Cultures were incubated at 37 °C in a humidified atmosphere with 5% CO_2_. Non-adherent cells were removed after 72 h and fresh medium was added, then the culture medium was changed every 3–4 days. At 80% confluence, MSC were washed with Dulbecco's phosphate-buffered saline (D-PBS, Sigma-Aldrich), harvested using 10X TrypLE Select (Gibco, Invitrogen) and sub-cultured at a density of 2 000 cells/cm^2^.

### 2.2 BM MSC characterization

#### 2.2.1 Immunophenotypic analysis

Cells at passage 4 were stained with anti-human antibodies against CD31-FITC (Clone 5.6E), CD45-ECD (Clone J.33), CD105-PE (Clone 1G2), CD90-FITC (Clone F15-42-1-5), CD44-FITC (Clone J.173) from Beckman Coulter (Fullerton, CA, USA) and CD34-PE (Clone 8G12), CD73-PC7 (Clone AD2) from Beckton Dickinson (Franklin Lakes, NJ, USA), 7-amino actinomycin D (7-AAD) from Invitrogen. Briefly, about 1 × 10^5^ cells were incubated for 15 min at room temperature (RT) with the specific antibodies (CD31/CD34/CD45/7AAD/CD73; CD90/CD105/CD45/7AAD, CD44/CD105/CD45/7AAD). After washing, at least 10 000 events were acquired using a FC500 flow cytometer (Beckman Coulter). Data were analyzed by Kaluza software 2.1 (Beckman Coulter).

#### 2.2.2 BM MSC trilineage differentiation

For osteogenic and adipogenic differentiation, BM MSCs at the end of passage 5 were seeded (2 000 cells/cm^2^) on coverslips arranged in 24-well plates (Falcon Corning, NY, USA) in the presence of growth medium. At 70–80% cell confluence, growth medium was replaced with differentiation medium that was renewed every 3–4 days for 21 days. Adipogenic differentiation was induced using the StemPro adipogenic differentiation kit (Invitrogen) according to the manufacturer's instructions. The presence of intracellular lipid droplets was detected by staining the cells with Oil Red O (Diapath, Martinengo, Italy). Osteogenic differentiation was induced using the StemPro Osteogenic differentiation kit (Invitrogen) according to the manufacturer's instructions. Fresh medium was added every 3–4 days for 21 days and the presence of calcium deposits was evaluated using von Kossa staining. Cells were fixed with 10% formalin for 5 min at RT, incubated with 1% silver nitrate solution (Sigma-Aldrich) for 15 min and exposed to ultraviolet light for 2 h. To remove unreacted silver, coverslips were rinsed with distilled water and 5% sodium thiosulfate (Sigma-Aldrich). Finally, cells were counterstained with Nuclear Fast Red Solution (Sigma-Aldrich). To induce chondrogenesis, 25 × 10^4^ cells were placed in a 15 ml tube and washed in order to form a pelleted cellular micromass at the bottom of the tube. The cell pellet was cultured in 500 μl of chondrogenic induction medium (StemPro chondrogenic differentiation kit, Invitrogen), following the manufacturer's instructions. Fresh medium was added every 3–4 days and after 28 days the micromass was fixed, embedded in agar, cut with a microtome, stained with Alcian Blue and counterstained with Nuclear Fast Red Solution (Sigma-Aldrich).

### 2.3 Flow cytometric quantification of allogeneic BM MSC lysis by PB MNC co-cultures

Target BM MSCs (n = 13) were resuspended at a concentration of 500 000 cells/ml in D-PBS, 5% FBS and 5mM EDTA to prevent cell to cell adhesions and to minimize cluster formation and doublet detection during cytometer acquisition. Hundred μl of this solution (50 000 target cells) were transferred to four 12 × 75 mm tubes.

Effector resting PB MNCs collected from a healthy volunteer were obtained by density gradient centrifugation (ρ = 1.077 g/ml, Lymphoprep, Stemcell Technologies, Vancouver, BC) of whole peripheral blood diluted 1:1 with phosphate-buffered saline (D-PBS, SIGMA). To investigate the MSC-related variability, we have challenged 13 mesenchymal batches against a single effector donor cell.

PB MNCs were collected from the interphase, washed twice with D-PBS and added to three test tubes containing target cells at ratios of 5:1, 10:1 and 30:1 PB MNC:BM MSC (E-T) in a 200μl final volume. Cells were co-cultured for 4 h in humidified atmosphere at 37 °C and 5% CO_2_. To quantify spontaneous cell death at the beginning (T = 0 h) and at the end of co-culture (T = 4 h) target cells were incubated alone (control tubes).

### 2.4 Cell staining

At the end of co-culture experiments, cells were stained for 15 min at RT in the dark by adding 10μl of CD45-ECD and 5μl of CD105-PE antibodies and 1μl of 7-AAD.

7-AAD is a nucleic acid dye solution for the exclusion of non-viable cells. Fluorescence is detected in the far red range of the spectrum (650 nm long-pass filter).

To obtain absolute cell counts 200μl of Flow Count Fluorospheres (Beckman Coulter) were added to the tubes. Flow Count Fluorospheres are a suspension of fluorescent microbeads. Each fluorosphere contains a dye which has a fluorescent emission range of 525nm–700nm when excited at 488nm.

Cell analysis was performed on CYTOMICS FC500 equipped with 488 nm and 633 nm laser. Before cell analysis, alignment and fluidics were checked by using Flow Check Pro beads (Beckman Coulter) and electronic compensations were adjusted by running individual cell populations stained with each dye. CD105-PE was detected in FL2 (575nm), 7AAD in FL4 (675nm), CD45-ECD in FL3 (610nm) and Flow Count Fluorospheres in FL1 (525nm) channels.

5 000 CD45- CD105 + target events were acquired

### 2.5 Gating strategy

Cytotoxicity, quantified using the KALUZA 1.2 software, was expressed by using absolute cell counts, as percent reduction of live BM MSCs between the control and the co-cultured cells (Figure 2). The fluorospheres, identified by their distinctive scatter, and the BM MSC debris, recognized by using a tube containing only BM MSCs (control tube), were excluded from the analysis. BM MSCs were identified as 7AAD-/CD45-/CD105+ and quantified as follows:

Concentration of MSCs = [(MSC events/ bead events) x calibration factor]

(The calibration factor was the bead concentration as declared by the producer).

The percentage of cell lysis was expressed as follows:

{[(concentration of MSCs alone - concentration of MSCs in co-culture)/concentration of MSCs alone] x100}

We compared this gating strategy with the most commonly used in which the cytotoxic activity of the effector cells was expressed as a percentage of dead cells in the overall target cell population. After having electronically discarded cell debris and having identified MSCs as CD45-/CD105+, the percentage of 7AAD+ BM MSCs was quantified. The percentage of dead BM MSCs was calculated by subtracting the percentage of dead BM MSCs in the control (incubated without PB MNCs) from the percentage of 7AAD+ BM MSCs incubated with effector cells.

### 2.6 Statistical analysis

The differences among group means were compared by one-way analysis of variance (ANOVA)-Newman-Keuls Multiple Comparison Test using the GraphPad Prism software Version 5.01 (GraphPad Software, Inc. La Jolla, USA). Probability (p.values) < 0.001 were considered as significant.

## 3 Results

### 3.1 Characterization of BM MSC

**Figure 1:**
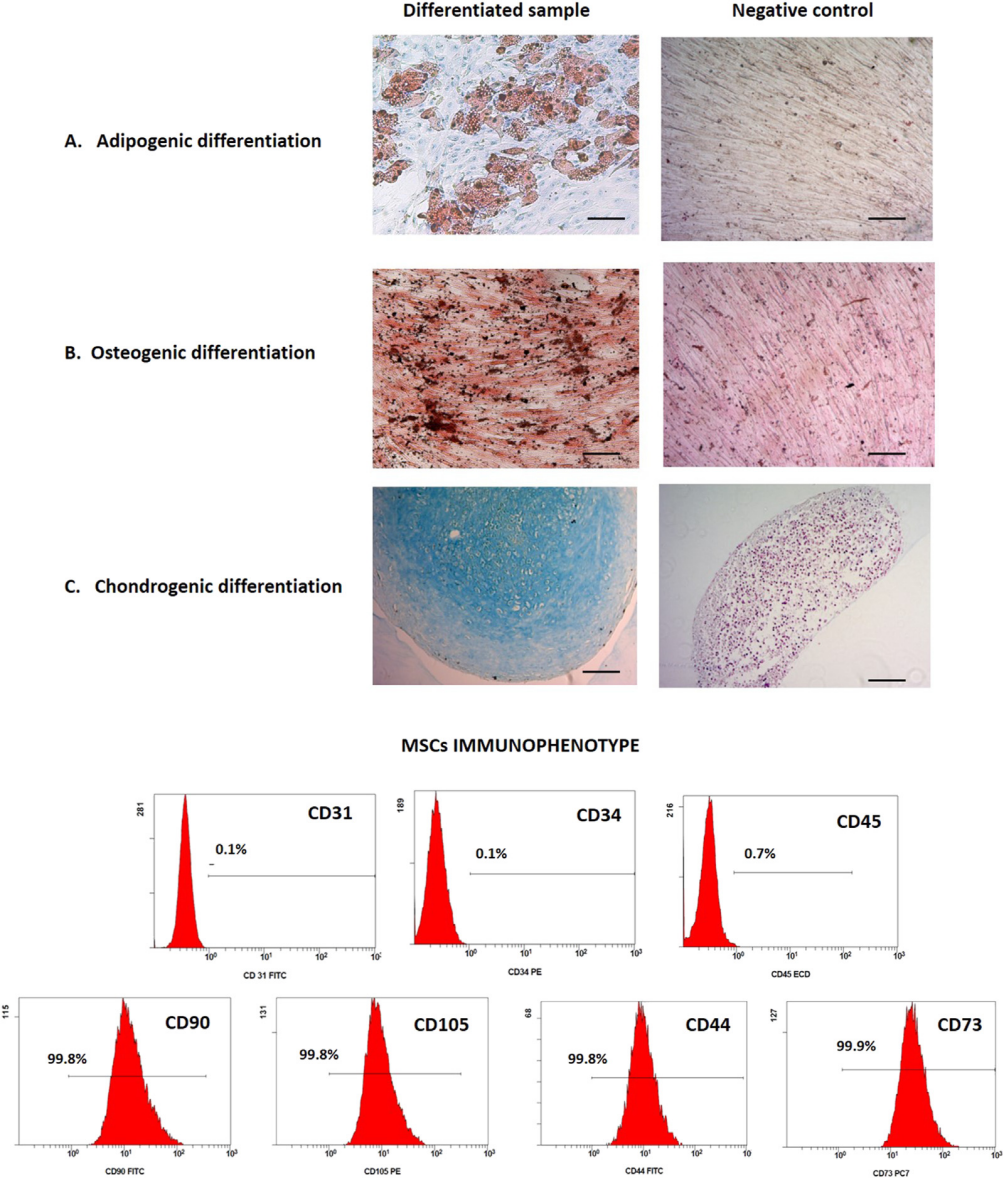
BM MSCs trilineage differentiation and immunophenotyping characterization of MSC. The presence of intracellular lipid droplets was detected by staining the cells with Oil Red O (A) and the calcium deposits using von Kossa staining (B); Alcian Blue stain showed the proteoglycans in the micromass (C). One representative case was reported, magnification 100X (Scale bar = 100 μm). Expression of CD31, CD34, CD45, CD90, CD105, CD44, CD73 on living (7AAD-) MSC. Unstained cells were used as gating controls.

BM MSCs had a spindle-shaped morphology, adhered to plastic, differentiated into the three mesodermal lineages and exhibited a characteristic MSC immunophenotype with positive expression of CD105, CD44, CD90, CD73 (between 97.0% and 99.9%) and the lack of CD45, CD31 and CD34 (between 0.1% and 0.7%) quantified on living MSCs discriminated using 7AAD (Figure 1).

### 3.2 Quantification of the BM MSC lysis by PB MNCs

**Figure 2:**
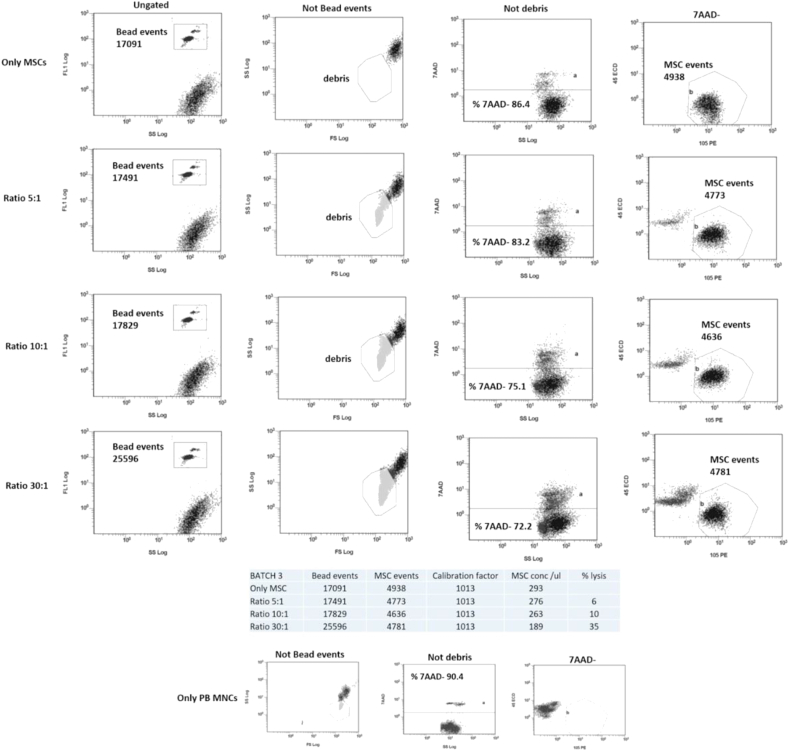
Gating strategy for the absolute cell count method. A calibration region was created to measure the bead signal. The debris gate consists of the apoptotic bodies of the BM MSCs derived from the lysis induced by the PB MNCs, the apoptotic bodies due to the spontaneous cell death of both BM MSC and PB MNC and the live PB MNCs which due to their small size, overlap with the BM MSCs debris. After discarding debris, the 7AAD- events were identified displaying fluorescence against SS. 7AAD- BM MSC were quantified by plotting CD45 versus CD105 and with the addition of fluorosphere beads at known concentration. The cytotoxic effect of PB MNCs on BM MSCs (batch 3) is reported at the different ratios (5:1, 10:1 and 30:1). PB MNCs and BM MSCs cultured alone were used as controls.

The gating strategy based on absolute cell count revealed that 6/13 (46%) of the BM MSCs were susceptible to PB MNC lysis. In 4/6 susceptible samples the lysis was above 15% at the highest ratio. In addition, the absolute cell count showed that the percentage of BM MSC lysis resulted to be directly proportional to the E/T ratio (Figures 2 and 4A), indicating that the assay discriminates different levels of cytotoxic activity, and significant between ratios 5:1–30:1 and 10:1–30:1 (p < 0.001. Figure 4B).

**Figure 3:**
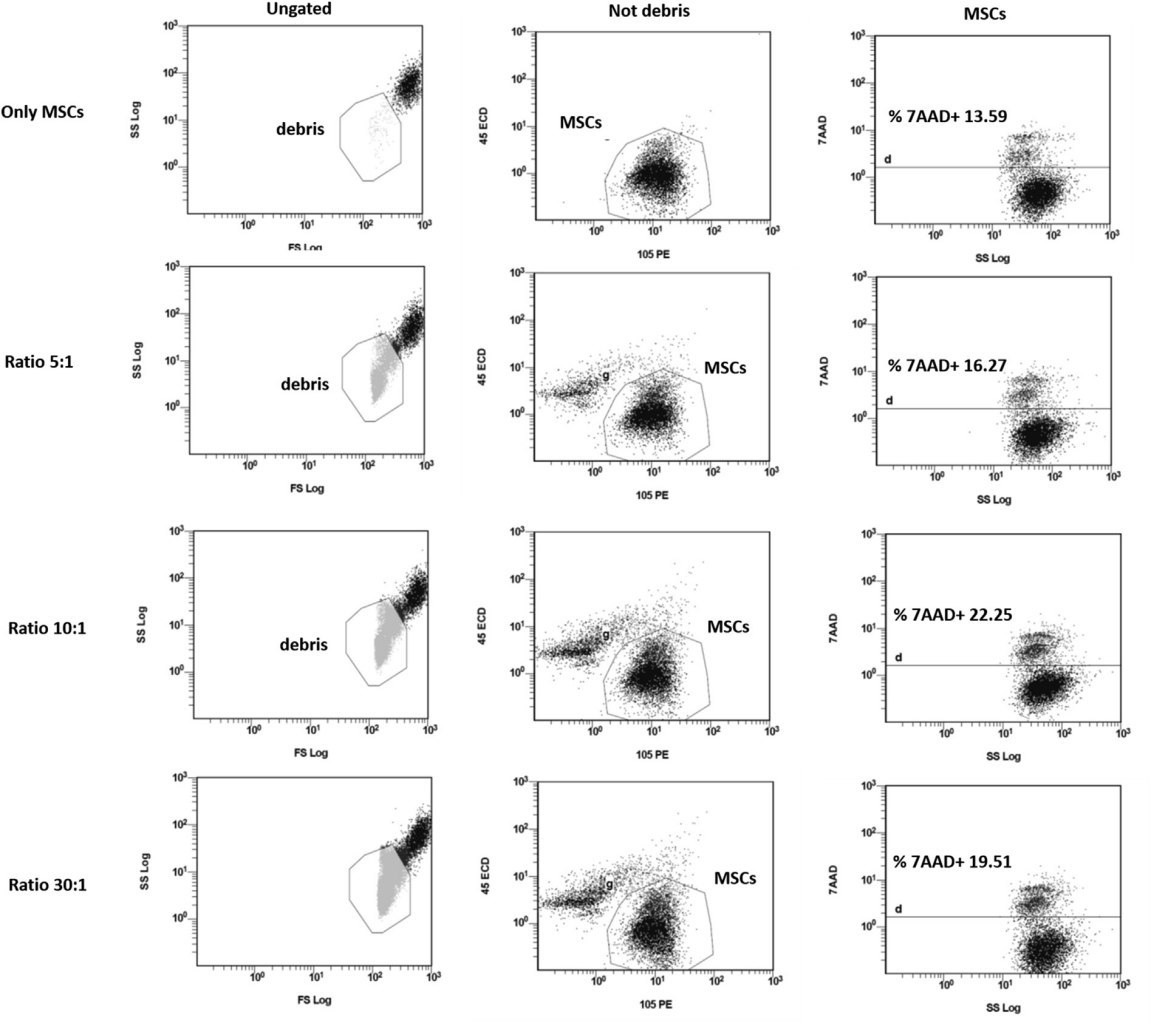
The cytotoxic activity of the effector cells is expressed as the percentage of dead BM MSC (batch 3) in the overall target cell population. After electronically discarding cell debris and identifying BM MSC as CD105+ and CD45-, the percentage of 7AAD + BM MSC was quantified (non-absolute cell count). The cytotoxic effect was underestimated when this gating strategy was applied.

**Figure 4:**
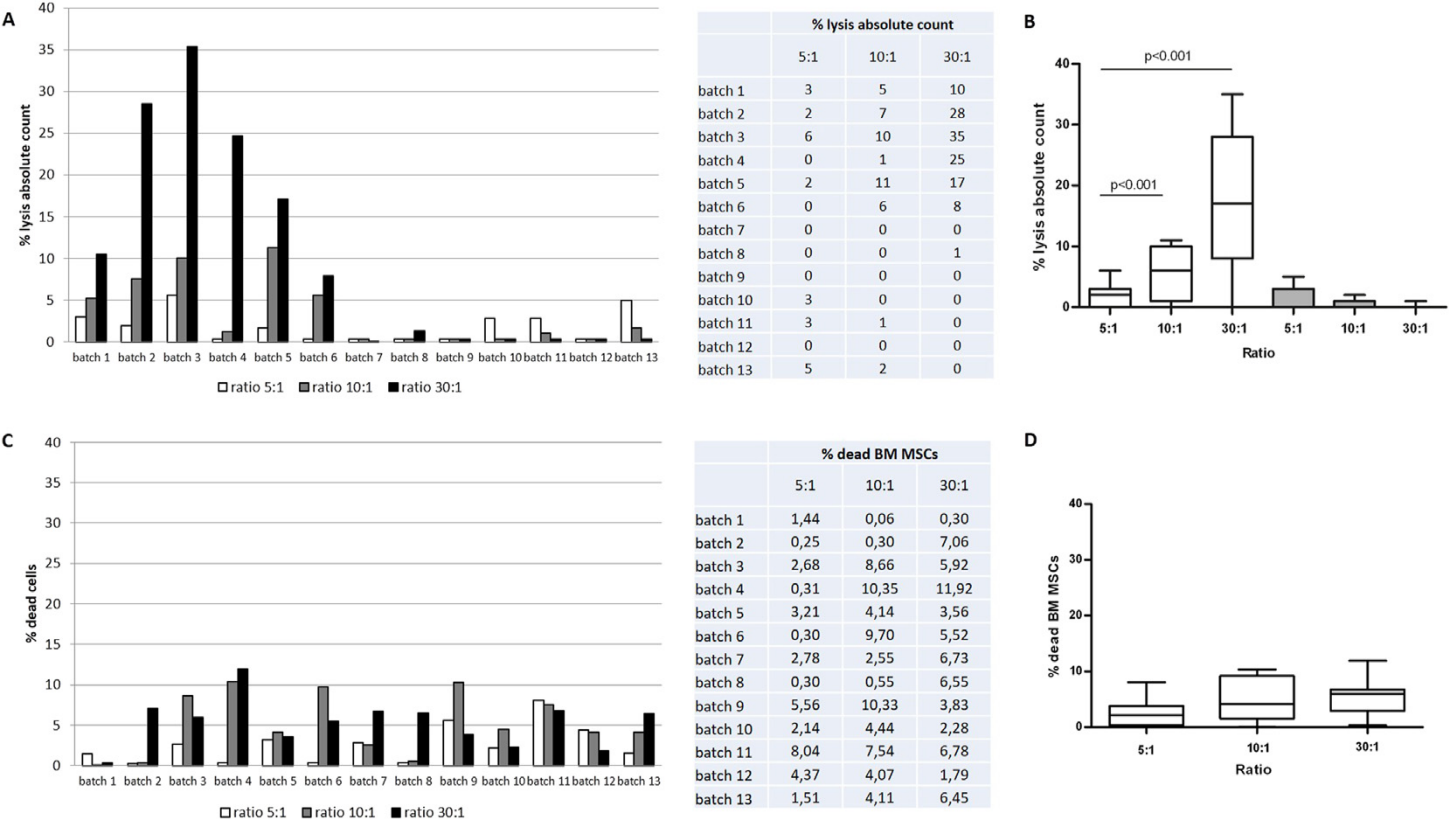
Percentage of lysis of BM MSCs (n = 13) induced by PB MNCs after 4 h of co-culture at E:T ratio of 5:1, 10:1 and 30:1 calculated by using: **A and B.** Absolute cell counts (column plot and box-whisker plot, white boxes batches 1–6, grey ones batches 7–13); **C and D.** Non absolute cell counts (column plot and box-whisker plot). This could be caused by the destruction of target cells that are no longer detected in flow due to the clearance of apoptotic bodies/debris in the analysis. In particular, the percentage of dead cells could be low especially at the highest ratio (30:1) masking the cytotoxic effect and resulting in a false negative.

Conversely, none or very low cytotoxic effect was measured when the percentage of dead BM MSCs in the overall target cell population was quantified. Indeed, none of the samples showed BM MSC lysis above the 15% threshold, even at the highest E/T ratio (Figures 3, 4C and D).

## 4 Discussion

Cytotoxicity assays have been classically performed by quantifying the number of dead target cells or by evaluating the effector activity in co-culture experiments.

The use of Chromium 51 (^51^Cr) has long been considered the reference method to quantify cytotoxicity. It is based on the estimation of the amount of ^51^Cr released in the culture media after cell lysis. The main drawback is the spontaneous release of the radioisotope resulting in an inter-assay variability of about 20%, particularly evident at low effector/target cell ratio. Moreover, radioisotopes must be manipulated in dedicated handling facilities. For this reason, the ^51^Cr release assay has been applied to MSCs in limited number of studies [14].

Calcein AM [20] is converted into calcein inside the cells whereas in presence of a cytolytic process it spreads into the supernatant. Similar to the ^51^Cr release assay, when a calcein release occurs spontaneously, the test can no longer be interpreted. In our experience, we have calculated that Calcein AM spontaneous release from BM MSCs is about 29% immediately after staining whereas it should not get over 5–8%, and after 24 h the efflux of the dye reached 91% (data not shown).

Lanthanide probes like Europium, works as calcein AM with the advantage that the fluorescence decay time is longer. This technology was used in a cytotoxicity assay with MSCs by Crop and collaborators [15], however the authors reported a spontaneous release of the probe of around 25%.

In conclusion, these probes are characterized by an unstable incorporation of the dye into MSCs that can cause a non-specific staining of the neighboring cells present in the assay. We observed that by using DIOC18 the unstained cells acquired the dye already after 4 h.

Another limitation of the dye-based assays is that they do not provide any quantitative information on the fate of the various cell subpopulations involved.

To overcome the described limitations, we developed an assay in flow cytometry based on differential immunostaining of target BM MSCs and effector PB MNCs in combination with a viability marker and fluorospheres for absolute count. This method leave the cells largely untouched, consequently cell losses occurring during washing steps that can influence the final result are avoided.

The quantification of cytotoxicity with absolute count showed that 46% of the MSC cell lots were susceptible to lysis in a ratio dependent manner. In about 31% of them the lysis was above the 15% threshold at the highest ratio (30:1) but the cytotoxic effect was underestimated (the percentage of lysis was always below 15%) by using an exclusion dye (7AAD). This because the apoptotic bodies derived from the lysed MSCs are removed from the analysis being considered debris and this affects the datum in particular at the highest E:T ratio in the presence of cell lysis.

As mentioned, the clinical use of MSCs has shown variable efficacy in the treatment of GvHD and only 50% of patients responds to therapy. Unfortunately, despite the large number of pediatric and adult patients affected by GvHD and regardless the huge industry investment in Phase III clinical trial, we still lack a predictive marker of clinical response.

Recently Dazzi and collaborators hypothesized that, to play their role, MSCs need to undergo apoptosis once in contact with the host's cytotoxic lymphocytes. Importantly, this hypothesis has been verified *in vivo* and has been demonstrated in GvHD patients where the activity against MSC correlates with the clinical response [29]. Notably, cytotoxicity activity against MSCs between clinical responders and non-responders was markedly different, and the 14.85% discrimination threshold of apoptotic MSCs was proposed as predictive of clinical response at E:T cell ratio of 20:1.

## 5 Conclusions

In the present work, we have developed a flow cytometric assay based on absolute cell counts. The implementation of the method is simple as long as a sample of patient peripheral blood is collected in advance, to be cultured with the MSC lot that will be infused. Since the percentage of MSCs lysis exerted by the same effector lymphocytes varied depending on the target MSCs, the assay could be proposed as a screening test for the identification of the most effective cell lot. Before drawing definitive conclusions on its real effectiveness, it is necessary to validate it in a controlled clinical trial.

## Declarations

### Author contribution statement

Giuseppe Astori: Conceived and designed the experiments; Analyzed and interpreted the data; Wrote the paper.

Katia Chieregato: Conceived and designed the experiments; Performed the experiments; Analyzed and interpreted the data; Wrote the paper.

Martina Bernardi: Performed the experiments.

Alberta Alghisi, Omar Perbellini, Mario Rassu: Analyzed and interpreted the data.

Rosaria Giordano, Lorenza Lazzari: Conceived and designed the experiments; Analyzed and interpreted the data; Contributed reagents, materials, analysis tools or data.

Marco Ruggeri: Analyzed and interpreted the data; Contributed reagents, materials, analysis tools or data.

### Funding statement

This study was supported by Associazione Vicentina per le Leucemie ed i Linfomi-Associazione Italiana Leucemie (AVILL-AIL).

### Data availability statement

Data will be made available on request.

### Declaration of interests statement

The authors declare no conflict of interest.

### Additional information

No additional information is available for this paper.

## References

[1] Amati E., Perbellini O., Rotta G., Bernardi M., Chieregato K., Sella S., Rodeghiero F., Ruggeri M., Astori G. (2018). High-throughput immunophenotypic characterization of bone marrow- and cord blood-derived mesenchymal stromal cells reveals common and differentially expressed markers: identification of angiotensin-converting enzyme (CD143) as a marker differentially expressed between adult and perinatal tissue sources. Stem Cell Res. Ther..

[2] Barilani M., Banfi F., Sironi S., Ragni E., Guillaumin S., Polveraccio F., Rosso L., Moro M., Astori G., Pozzobon M., others (2018). Low-affinity nerve growth factor receptor (CD271) heterogeneous expression in adult and fetal mesenchymal stromal cells. Sci. Rep..

[3] Pittenger M.F., Mackay A.M., Beck S.C., Jaiswal R.K., Douglas R., Mosca J.D., Moorman M.A., Simonetti D.W., Craig S., Marshak D.R. (1999). Multilineage potential of adult human mesenchymal stem cells. Science.

[4] Dominici M., Le Blanc K., Mueller I., Slaper-Cortenbach I., Marini F.C., Krause D.S., Deans R.J., Keating A., Prockop D.J., Horwitz E.M. (2006). Minimal criteria for defining multipotent mesenchymal stromal cells. The International Society for Cellular Therapy position statement. Cytotherapy.

[5] Di Nicola M., Carlo-Stella C., Magni M., Milanesi M., Longoni P.D., Matteucci P., Grisanti S., Gianni A.M. (2002). Human bone marrow stromal cells suppress T-lymphocyte proliferation induced by cellular or nonspecific mitogenic stimuli. Blood.

[6] Krampera M., Glennie S., Dyson J., Scott D., Laylor R., Simpson E., Dazzi F. (2003). Bone marrow mesenchymal stem cells inhibit the response of naive and memory antigen-specific T cells to their cognate peptide. Blood.

[7] Le Blanc K., Frassoni F., Ball L., Locatelli F., Roelofs H., Lewis I., Lanino E., Sundberg B., Bernardo M.E., Remberger M., others (2008). Mesenchymal stem cells for treatment of steroid-resistant, severe, acute graft-versus-host disease: a phase II study. Lancet.

[8] Le Blanc K., Tammik L., Sundberg B., Haynesworth S.E., Ringdén O. (2003). Mesenchymal stem cells inhibit and stimulate mixed lymphocyte cultures and mitogenic responses independently of the major histocompatibility complex. Scand. J. Immunol..

[9] Bartholomew A, Sturgeon C, Siatskas M, Ferrer K, McIntosh K, Patil S, Hardy W, Devine S, Ucker D, Deans R and others. Mesenchymal stem cells suppress lymphocyte proliferation in vitro and prolong skin graft survival in vivo. Exp. Hematol.;30:42-48. 10.1016/s0301-472x(01)00769-x 11823036

[10] Chen L., Tredget E.E., Liu C., Wu Y. (2009). Analysis of allogenicity of mesenchymal stem cells in engraftment and wound healing in mice. PloS One.

[11] Sun L., Akiyama K., Zhang H., Yamaza T., Hou Y., Zhao S., Xu T., Le A., Shi S. (2009). Mesenchymal stem cell transplantation reverses multi-organ dysfunction in systemic lupus erythematosus mice and humans. Stem Cells (Dayton, Ohio).

[12] Spaggiari G.M., Capobianco A., Becchetti S., Mingari M.C., Moretta L. (2006). Mesenchymal stem cell-natural killer cell interactions: evidence that activated NK cells are capable of killing MSCs, whereas MSCs can inhibit IL-2-induced NK-cell proliferation. Blood.

[13] Sotiropoulou P.A., Perez S.A., Gritzapis A.D., Baxevanis C.N., Papamichail M. (2006). Interactions between human mesenchymal stem cells and natural killer cells. Stem Cell..

[14] Götherström C, Lundqvist A, Duprez IR, Childs R, Berg L, le Blanc K. Fetal and adult multipotent mesenchymal stromal cells are killed by different pathways. Cytotherapy;13:269-278. 10.3109/14653249.2010.523077 20942778

[15] Crop M.J., Korevaar S.S., De Kuiper R., Ijzermans J.N.M., Van Besouw N.M., Baan C.C., Weimar W., Hoogduijn M.J. (2011). Human mesenchymal stem cells are susceptible to lysis by CD8+ T cells and NK cells. Cell Transplant..

[16] Chieregato K., Albiero E., Castegnaro S., Bernardi M., d'Amore E.S.G., Zanon C., Madeo D., Rodeghiero F., Astori G. (2012). A study on mutual interaction between cytokine induced killer cells Italy and umbilical cord-derived mesenchymal cells: implication for their in-vivo use. Blood Cells Mol. Dis..

[17] Eliopoulos N., Stagg J., Lejeune L., Pommey S., Galipeau J. (2005). Allogeneic marrow stromal cells are immune rejected by MHC class I– and class II–mismatched recipient mice. Blood.

[18] Zangi L., Margalit R., Reich-Zeliger S., Bachar-Lustig E., Beilhack A., Negrin R., Reisner Y. (2009). Direct imaging of immune rejection and memory induction by allogeneic mesenchymal stromal cells. Stem Cell..

[19] Poncelet A.J., Vercruysse J., Saliez A., Gianello P. (2007). Although pig allogeneic mesenchymal stem cells are not immunogenic in vitro, intracardiac injection elicits an immune response in vivo. Transplantation.

[20] Schu S., Nosov M., O'Flynn L., Shaw G., Treacy O., Barry F., Murphy M., O'Brien T., Ritter T. (2012). Immunogenicity of allogeneic mesenchymal stem cells. J. Cell Mol. Med..

[21] Lee R.H., Pulin A.A., Seo M.J., Kota D.J., Ylostalo J., Larson B.L., Semprun-Prieto L., Delafontaine P., Prockop D.J. (2009). Intravenous hMSCs improve myocardial infarction in mice because cells embolized in lung are activated to secrete the anti-inflammatory protein TSG-6. Cell Stem Cell.

[22] Ankrum J.A., Ong J.F., Karp J.M. (2014). Mesenchymal stem cells: immune evasive, not immune privileged. Nat. Biotechnol..

[23] Abdulrazzak H., Moschidou D., Jones G., Guillot P.V. (2010). Biological characteristics of stem cells from foetal, cord blood and extraembryonic tissues. J. R. Soc. Interface.

[24] Ciccocioppo R., Corazza G.R. (2016). Mesenchymal stem cells for fistulising Crohn's disease. Lancet.

[25] Le Blanc K., Rasmusson I., Sundberg B., Gotherstrom C., Hassan M., Uzunel M., Ringden O. (2004). Treatment of severe acute graft-versus-host disease with third party haploidentical mesenchymal stem cells. Lancet.

[26] Introna M., Rambaldi A. (2015). Mesenchymal stromal cells for prevention and treatment of graft-versus-host disease: successes and hurdles. Curr. Opin. Organ Transplant..

[27] Amati E., Sella S., Perbellini O., Alghisi A., Bernardi M., Chieregato K., Lievore C., Peserico D., Rigno M., Zilio A., others (2017). Generation of Mesenchymal Stromal Cells from Cord Blood: Evaluation of in Vitro Quality Parameters Prior to Clinical Use.

[28] Menard C., Pacelli L., Bassi G., Dulong J., Bifari F., Bezier I., Zanoncello J., Ricciardi M., Latour M., Bourin P., others (2013). Clinical-grade mesenchymal stromal cells produced under various good manufacturing practice processes differ in their immunomodulatory properties: standardization of immune quality controls. Stem Cell. Dev..

[29] Galleu A., Riffo-Vasquez Y., Trento C., Lomas C., Dolcetti L., Cheung T.S., von Bonin M., Barbieri L., Halai K., Ward S., others (2017). Apoptosis in mesenchymal stromal cells induces in vivo recipient-mediated immunomodulation. Sci. Transl. Med..

